# Erratum

**DOI:** 10.5152/tud.2025.252907

**Published:** 2025-07-29

On the declaration of interest sections of the articles titled “Unraveling Online Perspectives and Misinformation Surrounding Urinary Tract Infections: A Thematic Analysis of 1200 Instagram Posts”, “Minimally Invasive Surgical Approaches for Bladder Outlet Obstruction Within an Office Environment: A Comprehensive Literature Review” and “Reporting the Impact of Pelvicalyceal System (PCS) Anatomy on Clinical Outcomes in Retrograde Intrarenal Surgery (RIRS) Studies: Can We Do Better? – Methodological Review from the Section of EAU Endourology”; Bhaskar K. Somani’s Editorial Board Membership has errenously been omitted. Please refer to below links for the corrected articles:

 https://urologyresearchandpractice.org/en/reporting-the-impact-of-pelvicalyceal-system-pcs-anatomy-on-clinical-outcomes-in-retrograde-intrarenal-surgery-rirs-studies-can-we-do-better-methodological-review-from-the-section-of-eau-endourology-1611232

 https://urologyresearchandpractice.org/en/minimally-invasive-surgical-approaches-for-bladder-outlet-obstruction-within-an-office-environment-a-comprehensive-literature-review-1611231

 https://urologyresearchandpractice.org/en/unraveling-online-perspectives-and-misinformation-surrounding-urinary-tract-infections-a-thematic-analysis-of-1200-instagram-posts-1611230

